# A Novel Ex Vivo Model to Study Therapeutic Treatments for Myelin Repair following Ischemic Damage

**DOI:** 10.3390/ijms241310972

**Published:** 2023-06-30

**Authors:** Luisa Werner, Michael Gliem, Nicole Rychlik, Goran Pavic, Laura Reiche, Frank Kirchhoff, Markley Silva Oliveira Junior, Joel Gruchot, Sven G. Meuth, Patrick Küry, Peter Göttle

**Affiliations:** 1 Department of Neurology, Medical Faculty, Heinrich Heine University, 40225 Düsseldorf, Germanykuery@uni-duesseldorf.de (P.K.); 2 Molecular Physiology, Center for Integrative Physiology and Molecular Medicine, University of Saarland, 66424 Homburg, Germany

**Keywords:** ischemic stroke, neuroregeneration, myelin repair, oligodendrocyte

## Abstract

Stroke is a major reason for persistent disability due to insufficient treatment strategies beyond reperfusion, leading to oligodendrocyte death and axon demyelination, persistent inflammation and astrogliosis in peri-infarct areas. After injury, oligodendroglial precursor cells (OPCs) have been shown to compensate for myelin loss and prevent axonal loss through the replacement of lost oligodendrocytes, an inefficient process leaving axons chronically demyelinated. Phenotypic screening approaches in demyelinating paradigms revealed substances that promote myelin repair. We established an ex vivo adult organotypic coronal slice culture (OCSC) system to study repair after stroke in a resource-efficient way. Post-photothrombotic OCSCs can be manipulated for 8 d by exposure to pharmacologically active substances testing remyelination activity. OCSCs were isolated from a NG2-CreERT2-td-Tomato knock-in transgenic mouse line to analyze oligodendroglial fate/differentiation and kinetics. Parbendazole boosted differentiation of NG2^+^ cells and stabilized oligodendroglial fate reflected by altered expression of associated markers PDGFR-α, CC1, BCAS1 and Sox10 and GFAP. In vitro scratch assay and chemical ischemia confirmed the observed effects upon parbendazole treatment. Adult OCSCs represent a fast, reproducible, and quantifiable model to study OPC differentiation competence after stroke. Pharmacological stimulation by means of parbendazole promoted OPC differentiation.

## 1. Introduction

An ischemic stroke is caused by the acute disruption of blood supply. Treatment in ischemic stroke focuses on the early disease stages of lesion pathology, i.e., on restoring blood flow to the affected brain area (reperfusion). As time windows for reperfusion are narrow and treatment is sometimes not successful, persistent neurological deficits remain in the majority of patients. Disability is not only caused by neuronal and axonal degeneration but also by extensive loss of oligodendrocytes (OL) and their myelin sheaths [1]. Oligodendrocytes are highly vulnerable to ischemic damage and undergo necroptosis and apoptosis due to the release of toxic glutamate and ATP [2]. The latter, loss of oligodendroglial cells and their myelin sheaths (demyelination), was underestimated in the stroke field and mostly compromises axonal survival and correlates negatively with long-term clinical outcome and the disability degree [3,4]. Oligodendrocytes play a fundamental role in maintaining neuronal function and integrity through trophic and metabolic support [5,6]. Further studies indicated that remyelination is a major repair mechanism provoked by stroke-induced injury, whereby OPCs have a potential key function compensating for myelin loss and preventing additional axonal degeneration [7,8,9]. Herein, the infarct core represents an irreparably damaged area, as almost all cells die within a few minutes after occlusion. On the other hand, the peri-infarct area, termed penumbra, exhibits an area of brain tissue that is damaged but rescued either via restoration of blood flow (short-term) or myelin repair (long-term) [10,11,12,13]. In this regard, it is known that OPC activation, proliferation, and recruitment to the infarct region are induced [14], whereby an increased number of oligodendroglial cells are found in the penumbra, adjacent to the infarct core. Nevertheless, brain damage progresses at later stages of stroke, correlating with increased failure of OPCs to successfully differentiate into myelinating oligodendrocytes and leading to inefficient myelin repair [15,16,17]. Hence, a reliable stroke model to study oligodendroglial cell differentiation and maturation kinetics in the context of stroke is needed in order to investigate the effects of possible therapeutics to enhance the process of remyelination and restore normal brain function following stroke. To save resources and reduce animal suffering (3R principles), we set out to establish a slice culture model to test these substances ex vivo.

## 2. Results

**Figure 1:**
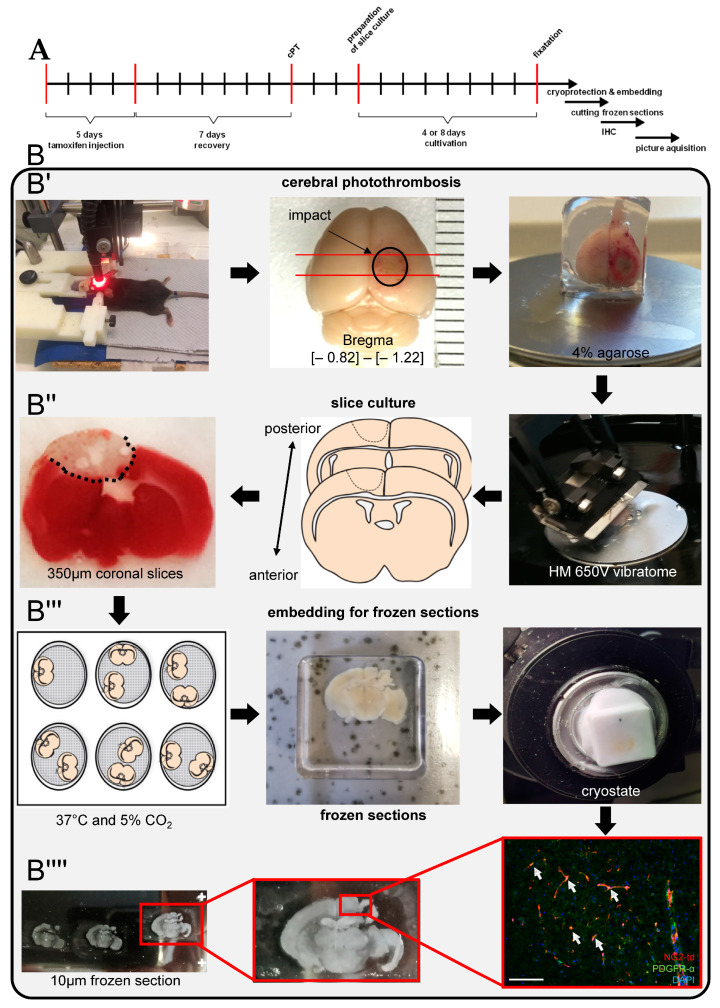
(**B**) Scheme of generating ischemic adult coronal slice cultures showing key steps in (**B′**) photothrombotic stroke, (**B″**–**B′′′′**) dissection process, and (**A**) timelines for slice culture protocol. (**B′′′′**) representative immunofluorecent staining, arrows indicating NG2+ cells positive for PDGFR-α. Scale bar (100 µM).

Within this study, we established a new ex vivo model of post-photothrombotic adult organotypic coronal slice culture (Figure 1) that provides an experimental design for the analysis of regenerative mechanisms of white matter damage after stroke. In terms of transferability, this ex vivo method is closer to the results of in vivo experiments compared to conventional in vitro cell culture studies, while at the same time, the number of animals compared to in vivo experiments is reduced. We used the transgenic NG2-CreERT2 td-Tomato knock-in mouse line [18] in order to pursue the response/fate of resident parenchymal oligodendroglial progenitor cells following stroke. NG2 (nerve/glia antigen-2) is a type I transmembrane glycoprotein (chondroitin sulfate proteoglycan 4) and expressed by resident parenchymal OPCs. This fraction of cells is widely accepted as a pool of resident precursor cells generating myelin-forming oligodendrocytes within adulthood that exhibit the potential target to promote cell-replacement therapies in the CNS [19,20].

### 2.1. Slice Culture Viability Following Photothrombotic Ischemia

**Figure 2:**
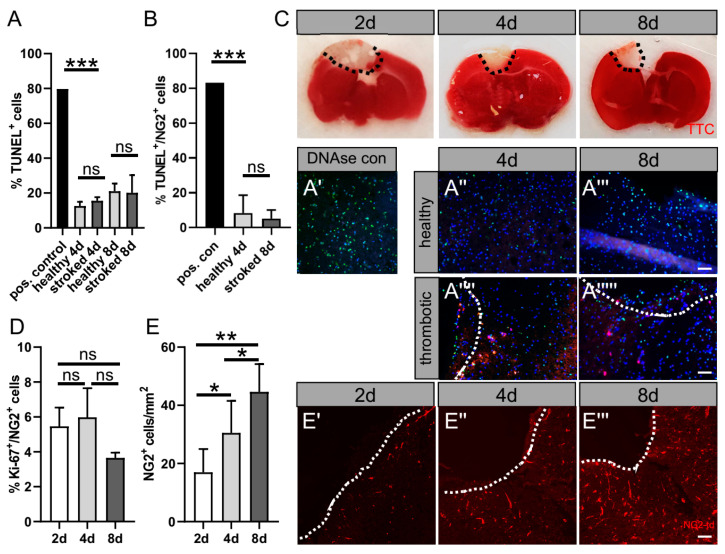
Representative data showing slice culture viability in culture post-photothrombosis. (**A**) TUNEL^+^ cells (**A′**) were counted and the percentage was calculated in relation to the total cell number assessed by DAPI^+^ cells for 4 d (**A″**,**A′′′′**) and 8 d (**A′′′**,**A′′′′′**). (**B**) Determination of apoptosis rate of NG2^+^ cells, double-positive cells for TUNEL and NG2 td-Tomato set in relation to the total number of NG2 td-Tomato^+^ cells. (**C**) Tissue viability following cerebral photothrombosis (cPT) is exemplified by 2,3,5-Triphenyltetrazolium chloride (TTC) staining on 2 d, 4 d, and 8 d in culture by the distinction between infarcted (no staining) and living tissue (red staining). Within the border zone following stroke, (**D**) the proliferation of NG2^+^ cells was determined by the quantification of Ki67 marker expression. (**E**) The number of NG2^+^ cells per mm within the border zone was quantified for (**E′**–**E′′′**) 2 d, 4 d and 8 d in culture. Dashed lines indicate the border between photothrombotically lesioned tissue and border zone. Scale bar: 50 µm. Data are shown as mean values (±SD) and are derived from (**A**,**B**) n = 3, (**C**) n = 5, (**D**) n = 10 experiments. Statistical significance was calculated using Tukey’s range test following one-way ANOVA (**A**–**E**). Data were considered statistically significant (95% confidence interval) at * *p* < 0.05, ** *p* < 0.01, *** *p* < 0.001, ns, not significant.

In the first step, we tested tissue viability by various means. A macroscopic indicator for healthy brain slices is the absence of cuts and increasing levels of transparency during cultivation [21,22,23]. Furthermore, we identified apoptotic cells within the cultivated slices in order to evaluate their viability during cultivation by using a TUNEL Assay (Figure 2A,B,A′–A′′′′).). Of note, our results showed no increase in apoptotic TUNEL-positive cells over time in cultivation, comparing slices cultivated for four or eight days. We also found no significant difference in apoptosis rates comparing cells within the hemisphere that underwent photothrombosis, excluding the ischemically lesioned area, and the contralateral, healthy hemisphere (Figure 2A). In addition, no differences in apoptosis rates of NG2^+^ cells at the border zone of the ischemic lesion over time (4 d–8 d) could be detected (Figure 2B). Moreover, we tested the viability of post-photothrombotic OCSCs following two, four, and eight days of cultivation by 2,3,5-triphenyl tetrazolium chloride (TTC) staining. The TTC is enzymatically reduced by living cells to a red dye produced by dehydrogenase [24]. This technique allowed us to check the overall viability of the organotypic slices after cultivation. Infarcted and damaged tissue stayed unstained while living tissue was stained red (Figure 2C) [24,25]. In general, our results suggest that the cells within the cultivated brain slices were mostly vital, with a moderate apoptosis rate under all conditions of below 20% (Figure 2). The analysis of the proliferation of NG2^+^ cells using the marker Ki-67 demonstrated no detectable differences over time, while proliferation was around 5% (Figure 2D). Interestingly, the number of NG2^+^ cells per mm^2^ adjacent to the lesion site increased over time (2 d–8 d). Together with the low proliferation rate, this may imply the migration of NG2^+^ cells to the border zone in the slices following stroke (Figure 2E).

### 2.2. NG2 Glia within the Border Zone of Ischemic Lesion

**Figure 3:**
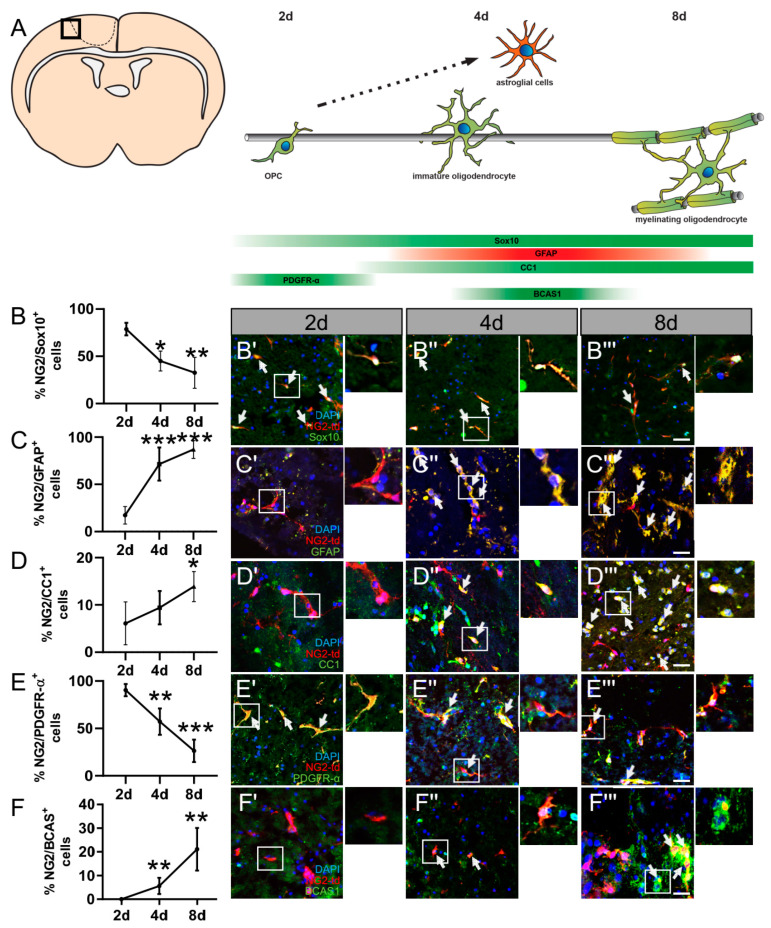
(**A**) Schematic overview of oligodendroglial marker expression kinetics within the border zone of the ischemic lesion by means of NG2-CreERT2-td-Tomato mice. (**B**–**F**) Quantification of confocal LSM micrographs within the border zone (**B′**–**F’’’**) of NG2 td-Tomato (red) positive cells coexpressing (**B**) Sox10, (**C**) GFAP, (**D**) CC1, (**E**) PDGFR-α, and (**F**) BCAS1 (green) under the border zone and within the lesion site 2 d, 4 d, and 8 d in culture. Arrows indicate double-positive cells. Dashed lines indicate the border between thrombotic core region and penumbra. Scale bar: 20 µm. Data are shown as mean values (±SD) and are derived from (**B**) n = 6, (**C**) n = 4, (**D**) n = 5, (**E**) n = 6, and (**F**) n = 7 experiments. Statistical significance was calculated using Kruskal–Wallis test with Dunn’s post-test (**B**) and Tukey’s range test following one-way ANOVA (**C**–**F**). Data were considered statistically significant (95% confidence interval) at * *p* < 0.05, ** *p* < 0.01, *** *p* < 0.001.

After the induction of td-Tomato expression in NG2^+^ cells by tamoxifen injection, one-week recovery and photothrombotic stroke as indicated in (Figure 1A), we analyzed the differentiation behavior/competence of NG2 glia at the border zone of the lesion site after 2, 4, and 8 days of cultivation. Given that the differentiation of adult NG2 glia in aged mice contributes to the generation of postnatal parenchymal OL lineage cells in the dorsal cortex and corpus callosum [18], we performed staining against different oligodendrocyte markers as well as the astrocytic lineage marker GFAP (glial fibrillary acidic protein) within the border zone of the lesion site (Figure 3A). Of note, almost all td-Tomato positive cells (tdT^+^) were restricted to the OL lineage according to the expression of OL lineage marker SRY (sex determining region Y) Sox10 at day 2 but decreased to 32.5% at day 8 in cultivation (Figure 3B). At the same time, the number of NG2^+^ cells coexpressing the astrocytic lineage marker GFAP significantly increased over time from only 17.3% at 2 d to 87% of cells being double-positive at 8 d (Figure 3C). In addition, we examined the differentiation competence of OPCs within OCSCs after 2, 4, and 8 d in culture by means of platelet-derived growth factor receptor alpha (PDGFR-α), adenomatous polyposis coli (APC) clone (CC1), and breast carcinoma amplified sequence 1 (BCAS1) immunostaining. At day 2, 90.5% of tdT^+^ in the border zone still expressed PDGFR-α, only expressed by immature OLs, whereas the percentage of PDGFR-α/tdT^+^-positive cells decreased to 26.3% at day 8 (Figure 3E). In accordance with the loss of PDGFR-α expression, NG2 glia were differentiating into OL and started to express mature OL differentiation marker CC1. Furthermore, 9.5% of tdT^+^ glia were already CC1^+^ at day 4, whereas at day 8, the percentage of CC1^+^/tdT^+^ glia significantly increased to 13.8%. Furthermore, BCAS1 was examined, as this marker is particularly suitable to identify newly formed myelinating oligodendrocytes and indicates regions where new myelin has been actively formed [26]. Our results show that at 4 days after cultivation, 5.6% of tdT^+^ glia were already BCAS1 positive cells, while the percentage of BCAS1/tdT^+^ significantly increased to 21.0% at d 8 within the border zone of the infarction (Figure 3F). These results demonstrate that NG2-expressing cells at the border zone of the lesion site are committed to the OL lineage and able to differentiate into myelinating OLs. However, these cells increasingly expressed an astrocytic lineage marker over time in culture. Taken together, these results show that the differentiation dynamics of NG2^+^ cells in OCSCs are comparable to known in vivo data [13,20].

### 2.3. Promotion of Oligodendroglial Differentiation within the Border Zone upon Parbendazole Exposition

**Figure 4:**
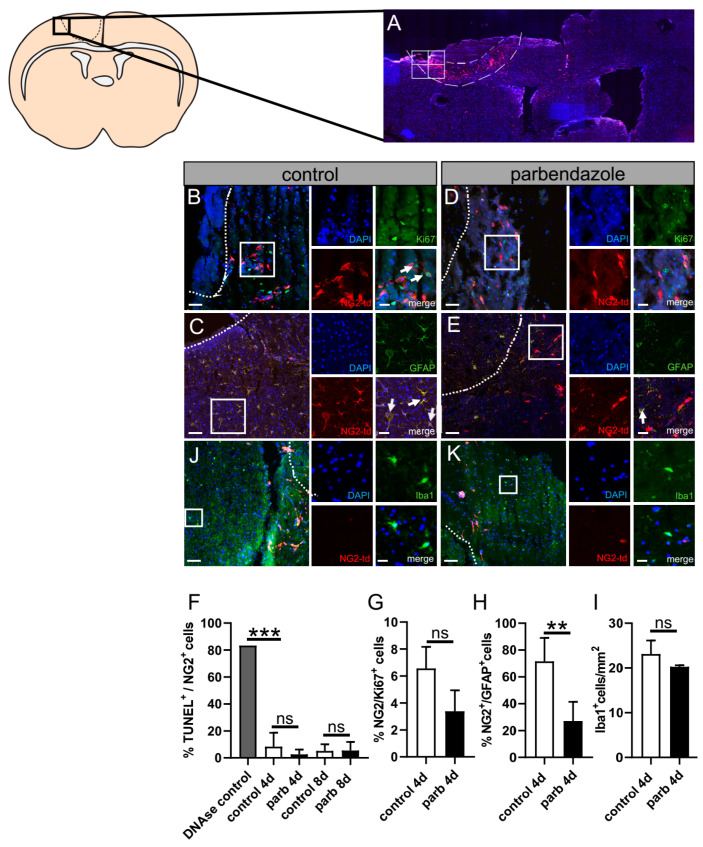
Effect of parbendazole on apoptosis, proliferation, microgliosis, and oligodendroglial cell fate of NG2^+^ cells within the border zone of post-photothrombotic adult coronal organotypic slice cultures. (**A**) Laser scanning micrograph (LSM) image of a photothrombotically lesioned NG2-CreERT2 td-Tomato mice brain after 4 days of cultivation. Immunostaining of cell nuclei using DAPI. The composition of 4 images (1 image measures 2195 × 1949 pixel or 482 × 428 µm) indicates the ROIs. Dashed lines indicate the border zone. Arrows indicate double-positive cells. (**F**) Quantification of NG2^+^ cells double positive cells for TUNEL indicating the apoptosis rate after 4 d and 8 d in culture. (**G**) Percentage of NG2^+^ and Ki-67^+^ double positive cells for DMSO control- (**B**) and parbendazole- (**D**) treated slice culture after 4 d. (**H**) Identification of astroglial lineage cells at the border zone of the photothrombotic lesion upon control (**C**) or parbendazole treatment (**E**) after 4 d. (**I**) Identification of Iba1 positive cells at the border zone upon control (**J**) or parbendazole (**K**) treatment after 4 d. Double-positive cells for NG2 td-Tomato and GFAP were counted and set in relation to the total amount of NG2^+^ cells. Dashed lines indicate the border between photothrombotically lesioned tissue and the border zone. Scale bars: (**A**) 10× magnification, (**B**,**D**) 50 µm, 20 µm, (**C**,**E**) 100 µm, and 20 µm, (**J**,**K**) 100 µm, 20 µm. Data are shown as mean values (±SD) and are derived from (**F**) n = 3, (**G**) n = 3, (**H**) n = 4, and (**I**) n = 3 experiments. Statistical significance was calculated using the Kruskal–Wallis test with Dunn’s post-test (**F**), and Mann–Whitney U test (**G**,**H**). Data were considered statistically significant (95% confidence interval) at ** *p* < 0.01, *** *p* < 0.001, ns, not significant.

**Figure 5:**
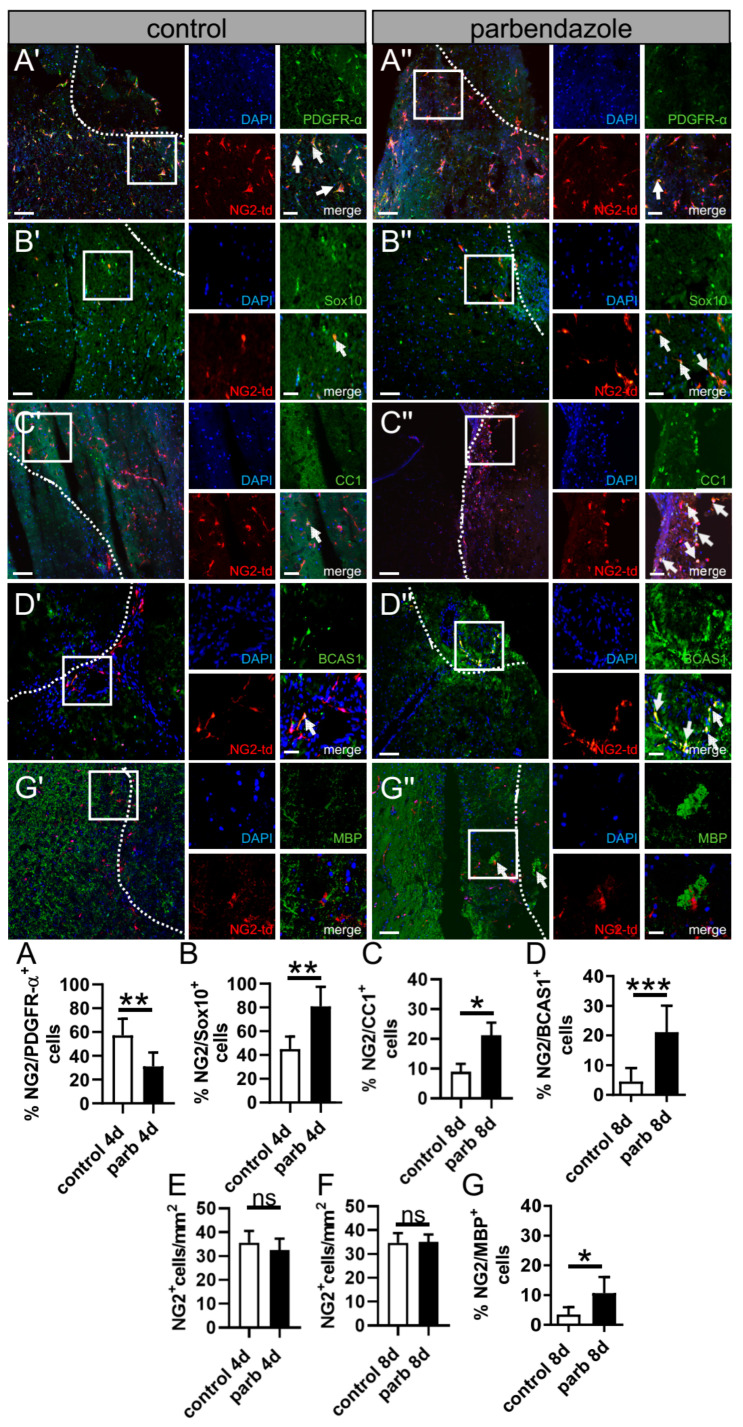
Promoting OPC differentiation within the border zone of post-photothrombotic adult coronal cultures following stimulation with parbendazole. (**A′**–**G″**) Representative images of oligodendroglial differentiation-associated protein markers on slices treated with parbendazole (**A″**–**D″**,**G″**) or DMSO, as control (**A′**–**D′**,**G′**). The impact on oligodendroglial cell differentiation and maturation was assessed by the number/mm^2^ of NG2^+^ cells and the relative percentage of double-positive cells (NG2^+^/PDGFR-α^+^ cells; (**A**–**A″**)), (NG2^+^/Sox10^+^ cells; (**B**–**B″**)), (NG2^+^/CC1^+^ cells; (**C**–**C″**)), (NG2^+^/BCAS1^+^ cells; (**D**–**D″**)), (NG2^+^/MBP^+^ cells; (**G**–**G″**)) along the same area. Arrows in detailed pictures indicate double-positive cells. Dashed lines indicate the border between ischemically lesioned tissue and the border zone. (**A′**–**G′′**) Scale bars: 100 µm, 20 µm. Data are shown as mean values (±SD) and are derived from (**A**) n = 7, (**B**) n = 5, (**C**) n = 7, (**D**) n = 7 and (**G**) n = 3 experiments. Statistical significance was calculated using Student’s *t*-test (**A**,**C**) and Mann–Whitney U test (**B**,**D**,**E**–**G**). Data were considered statistically significant (95% confidence interval) at * *p* < 0.05, ** *p* < 0.01, *** *p* < 0.001, ns, not significant.

Previous investigations using phenotypic compound screening revealed that, among other drugs, the anthelminthic drug parbendazole impacts OPC differentiation and myelin repair in the context of a cuprizone-mediated de(re)myelination mouse model for multiple sclerosis [27]. As parbendazole was shown, beyond its anthelmintic activity [28,29,30], to enhance OPC differentiation in the context of demyelination, we hypothesized that it could also show beneficial effects on remyelination after stroke. To this end, we used our new model (post-photothrombotic adult OCSCs) for stimulation experiments with parbendazole. To assess the effect of parbendazole, post-photothrombotic adult organotypic coronal slices were cultured with 0.02 µM parbendazole or DMSO as control for 4 or 8 days, respectively. Immunohistochemical staining was performed, to pursue the fate of NG2^+^ glia that migrated to the border zone of the photothrombotic lesion (Figure 4A). By use of anti-Ki67 staining, we could not detect a significant impact of parbendazole treatment on proliferation in NG^+^ cells at the infarction border zone at day 4 in culture (Figure 4B,D,G). To assess possible effects of parbendazole on apoptosis, a TUNEL assay was performed, showing no significant difference when OCSCs were treated with parbendazole (Figure 4F). Microgliosis is a dominant feature of stroke pathology impacting oligodendrogenesis; hence, Iba1 staining (Figure 4I–K) was performed. Herein, no differences in the distribution of microglia/myeloid cells positive for Iba1 upon parbendazole treatment could be detected. Given that OPCs have the potential to differentiate into oligodendrocytes or astrocytes [31,32] and an increment in the expression of GFAP was already seen in our OCSCs (Figure 3C), we were also interested in whether parbendazole exerts effects on astrocytes or the fate of OPCs. Therefore, GFAP staining was performed on the post-photothrombotic slice culture after 4 and 8 days in cultivation, and double-positive cells for tdT and GFAP at the border of the lesion were counted. Interestingly we found a high number of cells being double-positive (tdT^+^; GFAP^+^) under control conditions with 71.51%. This was significantly higher than in slices treated with parbendazole, with just around 27.11% (Figure 4C,E,H). These data suggest that parbendazole might stabilize the OPC lineage, rather than the astrocytic fate. To corroborate this finding, the state of oligodendrocyte differentiation was analyzed via immunofluorescence staining using anti-PDGFR-α antibodies, which are only expressed by immature precursors. Staining was performed on OCSCs cultivated for 4 days with or without parbendazole treatment. In addition, we conducted immunofluorescence staining using the late OL differentiation marker CC1 on slices cultivated to a maximum of 8 days to determine the number of further differentiated NG2 glia. Our results revealed that upon parbendazole treatment, the percentage of immature PDGFR-α positive NG2 cells was significantly lower at 31.1%, compared to control with 57.17% (Figure 5A). Correspondingly, we found that after 8 days of cultivation, the number of mature CC1-positive oligodendrocytes was significantly increased at 21.25% when comparing to control sections with 8.8% (Figure 5C). Consequently, the number of NG2/Sox10 double-positive cells increased from 45% to 81% (Figure 5B), and the number of NG2/CC1 cells increased from 8.9% to 21,2% (Figure 5C). Interestingly, our results also indicated a significant increase in the number of NG2/BCAS1 double-positive cells from 4.5% to 21.09% as well as of NG2/MBP double-positive cells from 3.5% to 10.6%, suggesting an increased remyelination potential. Of note, the overall cell number remained stable under parbendazole treatment (Figure 5E,F). Our data therefore indicate an enhanced oligodendroglial cell differentiation and enhanced potential for remyelination upon stimulation with the pharmacologically active compound parbendazole.

### 2.4. Characterization of Cells Invading the Scratch on Myelination Co-Culture

**Figure 6:**
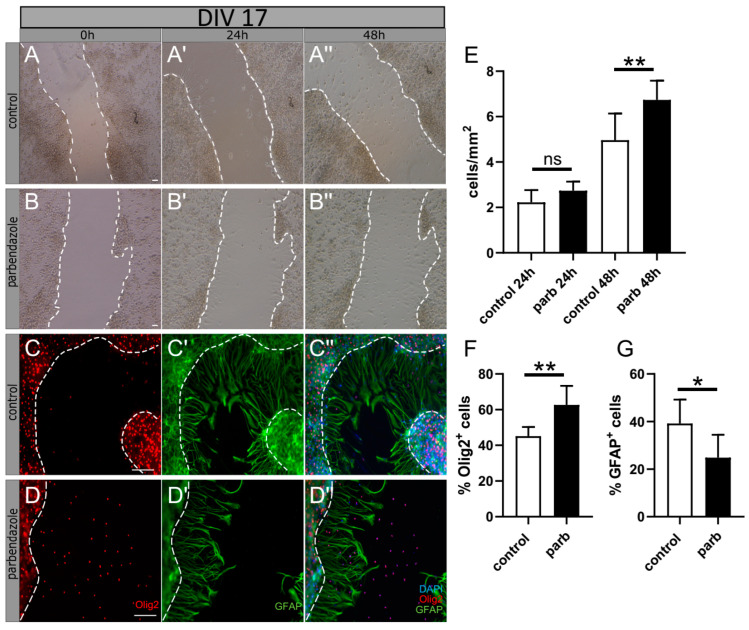
Identification and characterization of cells invading the scratch on myelination co-culture at 17 days in vitro (DIV). Representative bright field images of cells migrating into the scratch 24 and 48 h after stimulation with parbendazole (**B**–**B″**) and DMSO (**A**–**A″**) as control. Cells inside the scratch were counted, and the area of the scratch was calculated (**E**). Scale bar: 100 µm. After 48 h, the percentage of Olig2^+^ cells (**C**,**D**,**F**) and GFAP^+^ cells (**C′**,**D′**,**G**) was calculated in relation to the total number of cells, assessed by the number of DAPI^+^ cells, as exemplified by merged pictures (**C″**, **D″**). Scale bar: 100 µm. Data are shown as mean values (±SD) and are derived from n = 7 experiments for bright field images and n = 6 for immunofluorescence staining. Statistical significance was calculated using (**E**) Mann–Whitney U and Student’s *t*-test (**F**,**G**). Data were considered statistically significant (95% confidence interval) at * *p* < 0.05, ** *p* < 0.01, ns, not significant.

In order to reconfirm the effect of parbendazol on oligodendroglial’s fate in the context of injury, we performed a scratch assay on myelinating co-cultures at 17 days in vitro (DIV17), a timepoint where mature OLs start to myelinate axons in this culture system [33]. To this end, we used dissociated neuron–oligodendrocyte (OL) co-cultures from embryonic day 16 (E16) cerebral cortex. The co-culture was maintained in a modified myelination medium that supports both OL and neuron differentiation and comprises on average 38.5% NeuN+ neurons, 28.3% Olig2+ OL lineage cells, 10% of Glial fibrillary acidic protein (GFAP)+ astrocytes, and 10% CD11b+ microglia/macrophage at DIV10 [34]. To this end, cells that migrated into the scratch were counted after 0, 24, and 48 h and were then further characterized by immunofluorescent staining using the oligodendroglial lineage marker oligodendrocyte transcription factor 2 (Olig2) and the astrocytic marker glial fibrillary acidic protein (GFAP). We found that after 48 h, significantly more cells migrated into the scratch after parbendazole stimulation (Figure 6A,B,E). Quantification revealed that significantly more Olig2 positive cells migrated into the scratch, while the number of GFAP positive cells was significantly reduced compared to control conditions (Figure 6C,D,F–G). The same experiments were carried out on co-cultures at DIV24, a time point that is characterized by the onset of myelination. At this time, significantly more cells also migrated into the scratch after parbendazole stimulation; however, the ratio between Olig2 and GFAP positive cells only slightly and not significantly differed between the conditions. Hence, these data show that parbendazole is able to promote oligodendroglial cell migration at earlier time points.

### 2.5. Rescue of Oligodendroglial Differentiation Competence by Means of Parbendazole Treatment Following Chemical Ischemia after 3 d In Vitro

**Figure 7:**
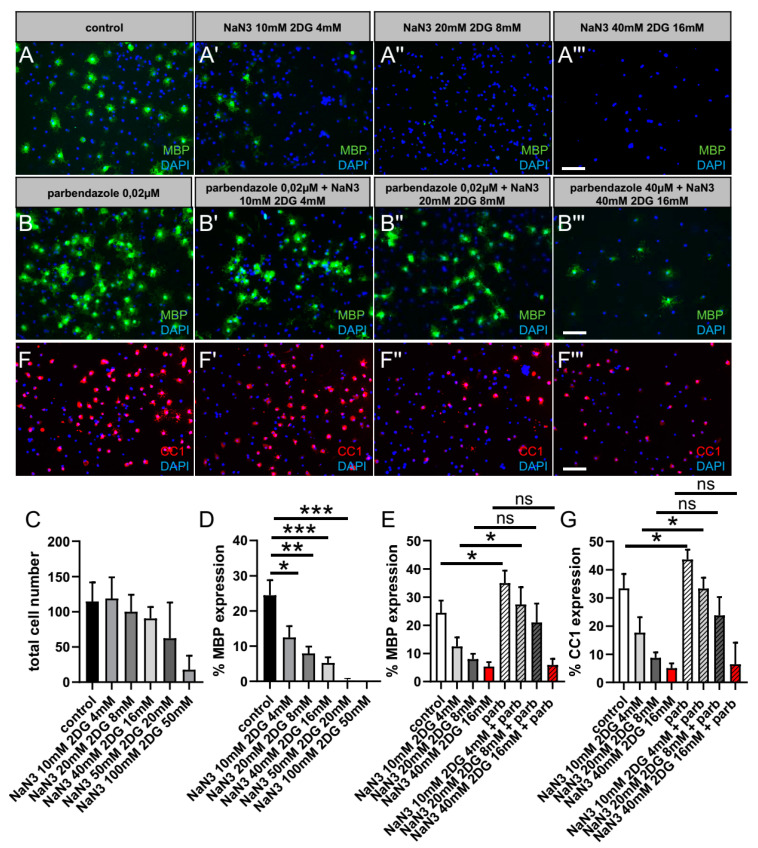
Rescue of oligodendroglial differentiation competence by means of parbendazole treatment following chemical ischemia after 3 d in vitro. Modification of oligodendroglial differentiation competence upon energy restriction removing glucose, blocking oxidative phosphorylation with sodium azide NaN_3_, and blocking glycolysis with 2-deoxy-D-glucose (2DG). In order to determine functionally relevant cell-specific concentrations, (**C**) total cell number as well as (**D**) MBP expression of primary cultured OPCs was investigated in a dose-dependent manner after 3 days in culture (**A**–**A′′′**). (**E**) In response to parbendazole treatment MBP (**B**–**B′′′**) as well as (**G**) CC1 (**F**–**F′′′**) expression can be rescued. Scale bar: 50 µm. Data are shown as mean values (±SD) and derive from n = 3 experiments. Statistical significance was calculated using Kruskal–Wallis test with Dunn’s post-test (**C**–**E**). Data were considered statistically significant (95% confidence interval) at * *p* < 0.05, ** *p* < 0.01, *** *p* < 0.001 ns, not significant.

In addition, our data were confirmed in a chemical ischemia model. We mimicked the effects of transient energy restriction in the ischemic penumbra by transiently removing glucose, blocking oxidative phosphorylation with sodium azide, and blocking glycolysis with 2-deoxy-D-glucose according to [35,36]. Those experiments were carried out on primary cultured OPCs, allowing us to confirm our data and investigate the effects of parbendazole directly on OLs, concerning energy deprivation, without other brain cells possibly interfering. To determine relevant cell-specific concentrations, total cell numbers as well as the expression of myelin basic protein (MBP), a marker that serves to monitor oligodendroglial differentiation, were investigated in a dose-dependent manner after 3 days in culture (Figure 7C,D,A–A′′′). Our data revealed that the total cell number is comparable to control conditions using concentrations ranging from 10 mM NaN_3_ with 4 mM 2DG up to 40 mM NaN3 with 16 mM 2DG. However, using higher concentrations, cell numbers were drastically reduced. Furthermore, immunofluorescence staining for MBP indicated a significant reduction in the expression of MBP with increasing NaN_3_ and 2DG concentrations. MBP expression was already diminished by about 50% using the lowest chemical ischemia concentrations, whereas MBP expression could not be detected using the highest concentration. Moreover, we investigated differentiation into mature oligodendrocytes by means of MBP (Figure 7E,B–B′′′) and CC1 (Figure 7G,F–F′′′) expression after stimulation with parbendazole. When cells were treated with parbendazole alone (without chemical ischemia), both MBP and CC1 expression was significantly higher compared to control conditions, confirming our findings above. Additionally, our data show that MBP as well as CC1 expression can be rescued by parbendazole stimulation after mild chemical ischemia (at low concentrations).

## 3. Discussion

This study demonstrates the capability of a new ex vivo model to study the differentiation kinetics of oligodendroglial cells within the peri-infarct region. Consistent with previous observations in demyelination models [27] and using two in vitro models, namely scratch assay and chemical ischemia, we here demonstrated that parbendazole treatment led to the accelerated generation of oligodendrocytes within the penumbra following stroke. Appropriate experimental animal models for cerebral ischemic damage in vivo such as cerebral photothrombosis or transient middle cerebral artery occlusion most closely reflect the molecular and cellular mechanisms after human stroke but encounter numerous challenges [37]. On the contrary, the 3R principles are guiding principles for more ethical use of animals “to avoid animal experiments altogether (Replacement), to limit the number of animals (Reduction) and their suffering (Refinement) to an absolute minimum” [38]. To this end, the ex vivo model of post-photothrombotic adult organotypic slice cultures established here offers the possibility of an experimental design for research on physiological, pathologic, and reparative mechanisms after cerebral damage following stroke that is close to animal studies but reduces the number of animals and their suffering and thereby addresses two Rs of the 3R principle. An experiment with n = 4 with two conditions and two time points requires at least 16 animals for experiments in vivo, whereas by means of our ex vivo model, significantly fewer animals (two animals) are required. At the same time, mechanisms can be studied more precisely in OCSCs than in vivo. OCSCs offer the opportunity to selectively and precisely add or remove cell populations, e.g., inflammatory cell populations, either by depletion paradigms or by modification of the time point for slice culture preparation, allowing or preventing the invasion of cells in vivo. They offer a greater temporal resolution (fixation/analysis at different time points within one probe) than in vivo models for fate mapping as exemplified for NG2^+^ cells over short time periods (8 d). Nevertheless, its needs to be taken into account that the short time window of tamoxifen induction and photothrombosis can result in anti-inflammatory and neuroprotective effects of this drug, as previously described [39]. Moreover, the lack of a blood–brain barrier improves control of drug concentrations and makes them appropriate for drug screening, e.g., parbendazole. Furthermore, live imaging analysis will be possible. The advantage compared to in vitro studies is the continued existence of tissue’s cell composition and connections in many respects. Nevertheless, slice culture thinning over time limits their temporal use in culture; hence, in vivo studies are more appropriate for long-term experimental investigation. Further limitations are that beside the analysis of mechanistic effects potential roles on disability, the clinical improvement and interaction of cells recruited in the in vivo situation from the organism after the time point of slice culture preparation cannot be assessed. Furthermore, emerging inflammation and trauma in OCSCs due to tissue slicing can generate a layer of astrocytic scar tissue onto the slice surface, altering mechanisms compared to the in vivo conditions [40]. As indicated above, this model has its limitations but can definitely serve as a prescreening platform for treatments, which can then be further validated in vivo, given that behavioral tests also cannot be assessed within this ex vivo model. With regard to brain injury, pathological analysis shows that demyelination or myelin loss is a main feature of ischemic stroke injury, suggesting that myelin repair is a major therapeutic target for functional recovery as demonstrated previously [11,12,13]. To this end, promoted myelin repair within the penumbra following stroke results in significant axonal regrowth and stabilization [41] and further improved neurological outcomes [3,8].

By means of NG2 td-Tomato knock-in mice, allowing us to monitor the fate, proliferation, migration, and differentiation of adult resident parenchymal OPCs, we were able to monitor the effect of the promyelination drug parbendazole on adult OPCs within the peri-infarct area following stroke. Of note, Sozmen and colleagues could demonstrate that the major response of OPCs after stroke arises from parenchymal progenitors as no recruitment of SVZ-derived cells in the OPC lineage post-stroke was found [13]. Given that oligodendroglial cell differentiation is inefficient in the environment of an ischemic lesion [17], the finding that parbendazole promotes myelin repair [27] in our ex vivo ischemic stroke model was of considerable interest. The differentiation kinetics shown in Figure 3 were boosted upon parbendazole treatment and enhanced oligodendroglial cell maturation, as indicated by alterations in PDGFR-α, CC1, Sox10, BCAS1, and MBP expression by NG2^+^ cells. Within this study, we focused on early time points and hence the generation of oligodendrocytes from OPCs and very early myelination indicators such as BCAS1 and MBP expression. Stretching the time in culture might give an idea of remylination. In the end, “the final aim” remyelination and the effect on the clinical score will have to be studied in vivo, which is behind the scope of this study. As parbendazole was administered within a pathophysiological scenario of ongoing inflammation, trauma, astrogliosis, and microgliosis, as well as OPC proliferation and migration, additional primary or secondary cytoprotective effects cannot be fully excluded. Given the restriction of Ki67 only providing a snapshot of proliferating cells at the very moment when slices were fixed, future studies regarding the role of proliferation during lesion expansion or drug treatment need to be performed by means of BrdU or EdU incorporation. However, we previously demonstrated OPC-specific effects of parbendazole in vitro and in vivo [27], and our current cell culture experiments (Figure 7) additionally suggested that OPC differentiation is the primarily targeted process. As resident OPCs are known to migrate towards the peri-infarct area before differentiating into mature oligodendrocytes [42], as indicated in Figure 2E–E′′′), it was interesting to assess the migration of adult OPCs upon parbendazole treatment. Whereas migration seems to play a role in NG2^+^ cell accumulation in the border zone of our slices over time, no difference in the number of NG2^+^ cells at the peri-infarct zone could be detected upon parbendazole treatment. In contrast, juvenile (developmental) OPCs, as demonstrated in the scratch assays of embryonal cells of the neuron–oligodendrocyte in vitro myelination culture (Figure 6), showed increased migration behavior after parbendazole treatment. Their higher resistance to ischemic damage compared to adult OPCs and higher affinity to respond to injury [43] might explain this difference. On the other hand, considering that slices were prepared 3 days after the induction of cerebral photothrombosis to allow inflammatory cells to invade the tissue, the initiation of OPC migration might be missed by the parbendazole treatment of the ex vivo slice culture [12].

The plasticity of oligodendroglial cells upon acute brain injuries can evoke various response cascades directing the conversion of oligodendrocytes into astrocytes [44].

In this context, it is interesting to mention that miconazole, another imidazole derivative related to parbendazole (a group of heterocyclic compounds that have biological, chemical properties to treat parasitic illnesses produced by either protozoa or helminthes [45]), was found to promote the neuroregeneration and neurobehavioral recovery of rats after t-MCAO via a brain-derived growth factor (BDNF)-dependent mechanism [46]. In addition, BDNF was demonstrated to maintain and stabilize oligodendroglial lineage in a dose-dependent manner [47]. This is in accordance with the finding that parbendazole reduces GFAP expression within NG2^+^ cells both ex vivo and in vitro (Figure 4H and Figure 6G). Nevertheless, to what degree parbendazole initiates the same pathway and whether, further cells beyond oligodendroglia can also be addressed by this drug need to be investigated in future experiments. Moreover, the results and interpretation presented here need to be further evaluated in other anatomic regions as well as different stroke models prior to translation to humans.

## 4. Materials and Methods

### 4.1. Ethics Statements for Animal Experiments

Cerebral-photothrombosis-induced cortical ischemia experiments were approved by the authorities at LANUV (Landesamt für Natur, Umwelt und Verbraucherschutz NordrheinWestfalen; Az.: 81-02.04.2018.A275) and carried out according to ARRIVE guidelines. The animals were bred under defined conditions with access to food and water ad libitum in the animal facility (ZETT) of the Heinrich-Heine-University Düsseldorf. For preparation of rat myelinating co-culture and OPC mono-culture experiments, cerebral cortices of Wistar rats of either sex were used—AZ.: 81-02.04.2018.A388. For adult organotypic cortical slice culture, ten- to twelve-week-old transgenic NG2-CreERT2 td-Tomato mice of either sex were used.

### 4.2. Mouse Line

Ten- to twelve week-old NG2-CreERT2 (Cspg4-CreERT2) td-Tomato mice generated as described elsewhere [18] were used for adult organotypic cortical slice culture 3 days after the induction of a cerebral photothrombosis. The NG2-CreERT2 knock-in mouse line, in which the open reading frame of the tamoxifen-inducible form of the Cre DNA recombinase (CreERT2) was inserted into the cspg4 (NG2; neural/glial antigen 2) locus via homologous recombination, was crossbred to a Rosa26-td-Tomato reporter line. In NG2-CreERT2/Rosa26-tdTomato mice, NG2 positive cells can be labeled and their fate map tracked in vivo upon induction of the Cre activity via tamoxifen. All mouse lines were maintained in the C57BL/6 N background, and only NG2 and reporter-heterozygous mice were used.

### 4.3. Oligodendroglial Cell Culture

Generation of primary OPCs from postnatal day zero (P0) cerebral rat cortices (Wistar rats of either sex) was performed as previously described [33]. The Institutional Review Board (IRB) of the ZETT (Zentrale Einrichtung für Tierforschung und wissenschaftliche Tierschutzaufgaben) at the Heinrich Heine University Düsseldorf has approved all animal procedures under licenses O69/11 and V54/09. Anti-A2B5 staining (Merck Millipore, Darmstadt, Germany; MAB312R RRID:AB_11213098) revealed that the cultures consisted of 98% oligodendroglial cells. OPCs were either seeded onto 0.25 mg/mL poly-D-lysine-coated (PDL, Sigma-Aldrich, St. Louis, MO, USA) glass coverslips (13 mm) in 24-well plates (for immunocytochemistry; 2.5 × 10^4^ cells/well) or onto 0.25 mg/mL PDL coated 24-well plates (for quantitative reverse transcription polymerase chain reaction (qRT-PCR); 5 × 10^4^ cells/well) in high-glucose DMEM-based Sato medium. After 1.5 h, oligodendroglial cell differentiation was initiated via Sato medium supplemented with 0.5% fetal bovine serum (FBS) (Capricorn Scientific, Palo Alto, CA, USA). The medium was exchanged every 3 days. To determine the physiological reaction from OPCs exposed to the substances, OPCs were treated in a dose-dependent manner using concentrations of 10 mM, 20 mM, 40 mM, 50 mM, and 100 mM for sodium azide (NaN_3_; Sigma-Aldrich, St. Louis, MO, USA Cat# S2002-100G); 4 mM, 8 mM, 16 mM, 20 mM, and 50 mM for 2-Deoxy-D-Glucose (2-DG; Sigma-Aldrich, St. Louis, MO, USA Cat# D8375-5G); and 0.02 µM for parbendazole (MedChem Express, Monmouth Junction, NJ, USA, CAS 14255879). The 0.02 µM application dosage was found to be the most effective compared to the others [27]. Stock concentrations of NaN_3_ (4.14 mM), 2-DG (10 mM) and parbendazole (10mM) were prepared using dimethyl sulfoxide (DMSO, Sigma-Aldrich, St. Louis, MO, USA) and this solvent was also used as control at equal dilutions. A 500 µL measure of prepared solutions was added to the cells containing either substances or DMSO, and final concentrations of substances were used.

### 4.4. Myelinating Co-Cultures

Dissociated neuron/oligodendrocyte co-cultures were obtained from embryonic day 16 (E16) rat cerebral cortices (Wistar rats of either sex) according to [33]. Cortical cells were plated on 15 mm poly-D-lysine (0.1 mg/mL) coated coverslips (65,000 cells per coverslip) and kept in myelination medium consisting of N2 and neurobasal medium (ThermoFisher Scientific, Waltham, MA, USA; ratio 1:1) including NGF (50 ng/mL) and NT-3 (10 ng/mL) (both R&D Systems, Minneapolis, MN, USA). The day of primary culture was defined as day one in vitro (DIV1). After 10 days in vitro (DIV10), insulin was excluded, and the ratio of the insulin-free N2 to neurobasal medium including the B27 supplement (ThermoFisher Scientific) was adjusted to 4:1. This myelination medium was further supplemented with 60 ng/mL tri-iodo-thyronine (T3, Sigma-Aldrich, St. Louis, MO, USA). Final concentrations of individual N2 medium components (DMEM-F12 based, high glucose; ThermoFisher Scientific) were insulin (10 μg/mL), transferrin (50 μg/mL), sodium selenite (5.2 ng/mL), hydrocortisone (18 ng/mL), putrescine (16 μg/mL), progesterone (6.3 ng/mL), biotin (10 ng/mL), N-acetyl-L-cysteine (5 μg/mL) (all Sigma-Aldrich St. Louis, MO, USA), bovine serum albumin (0.1%, Roth, Karlsruhe, Germany), and penicillin–streptomycin (50 units/mL, ThermoFisher Scientific). Co-cultures were kept for 17 days (DIV17) or 24 days (DIV24) in vitro, respectively, until a scratch assay was performed. During that time, the medium was exchanged every three days with the myelination medium.

### 4.5. Scratch Assay of Myelinating Co-Cultures and Stimulation

A scratch assay within the myelinating co-culture was performed on either DIV 17 or DIV 24 according to [48], with a few modifications. To this end, a 20 µL sterile pipette tip was used to generate a scratch on the cell surface from the upper part of the cover slip vertically down to the lower part of the cover slip. During this procedure, constant speed, 60–80° angle and moderate pressure were kept equal to gain comparable scratches with a similar width on all coverslips. Directly afterward, the scratch medium was exchanged with the new medium additionally containing 0.02 µM parbendazole or DMSO as control. Afterward, pictures of the scratch were taken 3 days (t1 = 0 h, t2 = 24 h, t3 = 48 h) in a row until fixation. Pictures were taken using the Nikon eclipse TE200 microscope in 20× magnification at the upper and the lower edge of the scratch. In between the picture acquisition, cells were placed back into the incubator at 37 °C, 5% CO_2_.

### 4.6. Tamoxifen Administration

To generate NG2-positive cells in the tissue, tamoxifen (10 mg/mL, T5648, Sigma-Aldrich, St. Louis, MO, USA) dissolved in corn oil (Sigma-Aldrich, St. Louis, MO, USA) was administered (100 mg/kg body weight) intraperitoneally once per day for 5 consecutive days in order to induce Cre activity according to [18].

### 4.7. Induction of Cerebral Photothrombosis (cPT)

After tamoxifen injection, NG2-CreERT2 td Tomato mice had 1 week of recovery in order to give cells time to successfully express the red fluorescent protein td-Tomato in NG2-cells/OPCs, before the introduction of cerebral photothrombosis. Cerebral photothrombosis was induced according to [21]. Briefly, a fiber optic bundle coupled to a cold light source (Schott EL 1500, Mainz, Germany) was centered 2 mm posterior and 2.4 mm lateral from Bregma. After the intraperitoneal injection of 1 mg Rose Bengal (Sigma Aldrich, St. Louis, MO, USA), the brain was illuminated through the intact skull for 15 min. The dye was photochemically activated and caused sendothelial damage and thrombosis in the brain supplying vessels in the light cone and thus resulted in a circumscribed cerebral infarction. After the illumination was stopped, the skin was readapted using tissue adhesive and the animal was allowed to wake.

### 4.8. Preparation of Organotypic Coronal Slice Culture (OCSCs)

In general, the entire procedure from slice generation and slice transfer to culture should take less than one hour in order to reduce stress and preserve viability. Briefly, eleven-week-old NG2-CreERT2 td-Tomato mice were used for OCSCs which were prepared 3 days after induction of cerebral photothrombosis (cPT) (Figure 1). The induction of td-Tomato in NG2^+^ cells was performed via intraperitoneal injection of 0.1 mL tamoxifen per day/animal for five days to induce Cre activity according to [18]. After one week of a recovery phase, cPT was performed according to [21], inducing a well-reproducible, stereotactically targeted ischemic damage by means of photo-activation of a previously injected photoactivable dye through the intact skull. Three days after the induction of cerebral photothrombosis, adult organotypic slice cultures (350 µm) were generated, adapted from [49]. Mice were deeply anesthetised by means of isoflurane, decapitated, and their brain was removed. To remove blood and hair from the brain, the brain was washed with ice-cold sterile Hank’s balanced salt solution (HBSS) (Gibco, Waltham, MA, USA Cat# 14025-050). The cerebellum and the brain stem were removed with a scalpel. Under aseptic conditions, the remaining brain was embedded in 4% low-melting-point agarose (Sigma-Aldrich Ca# A9414) in plastic embedding molds (Sakura, Umkirch, Germany, Tissue-Tek Cryomold Intermediate 15 mm × 15 mm × 15 mm Cat# 4566). The embedding mold was placed on ice until the agarose became solid. Thereafter, the agarose brain was glued to the magnetic platform of the HM 650 V vibratome (Thermo Scientific, Waltham, MA, USA) and was submerged in a chamber filled with ice-cold HBSS. The brain was trimmed until the stroke lesion occurred—Bregma [−0.82]–[−1.22]. Then, brain sections were cut with a thickness of 350 µm (cutting frequency: 50 Hz, cutting amplitude: 1.0, cutting speed: 0.9 mm/s). Slices were collected in a Petri dish with ice-cold HBSS, and then they were placed on cell culture inserts. From each brain, 8 slices were obtained. After two, four, or eight days of cultivation, the slices were fixed with 4% PFA and embedded for cryo-sectioning, allowing to quantify the contribution/differentiation dynamics of NG2^+^ cells via serial immunohistochemical staining per slice (Figure 1).

### 4.9. Slice Culture Conditions and Stimulation

Millicell cell culture inserts measuring 0.4 µm (hydrophilic PTFE, pore size: 0.4 µm, diameter 30 mm) (Millipore, Tullagreen, Carrigtwohill, Ireland Ref# PICM0RG50) were transferred under sterile conditions into a 6-well plate. Prior to the preparation of the slices, inserts were covered with 1.2 mL serum-supplemented medium (SSM, Table 1), and the membrane was incubated for 1 h in the incubator at 37 °C and 5% CO_2_. This equilibration step ensures the membrane’s alignment with media humidity, temperature, and pH. After a short washing step in SSM, brain slices were transferred on the membrane of Milicell cell culture inserts, with 2 slices per insert, using a spatula, and inserts were placed onto 1 mL of SSM medium. Excess medium on top of the membrane was removed to guarantee an air–medium interface for proper oxygenation and attachment to the slice [50]. They were then incubated with the SSM medium for 2 h at 37 °C and 5% CO_2_ in order to confer recovery from mechanical injury. After the recovery period, the medium was changed to serum-free medium (SFM, Table 1), given SFM’s beneficial properties for neuronal and glial viability, as well as the structural integrity of adult organotypic slice cultures [49,51,52]. SFM medium of slices was supplemented with 0.02 µM parbendazole (10 mM stock, MedChem Express Monmouth Junction, NJ, USA, CAS 14255-87-9) or DMSO as control. The parbendazole stock concentration (10 mM) consisted of 13.33 mg/mL in DMSO, which was further diluted in SFM. The slices were cultured to a maximum of 8 days at 37 °C and 5% CO₂. The medium was exchanged every 2 days.

### 4.10. TTC Staining

To show the viability of organotypic slices following eight days of cultivation, 2,3,5-Triphenyltetrazolium chloride (TTC) stainings were performed to allow the differentiation between infarcted (no staining) and living tissue (red staining). TTC indicates tissue viability and reveals the infarct size [24,25] (Figure 1). After 8 days of cultivation, organotypic slice cultures were incubated for 30 min in a 1% TTC solution at 37 °C. The TTC is enzymatically reduced by living cells to a red dye produced by dehydrogenase. For the TTC solution, 8 parts of 1.4% di-sodium-hydrogen phosphate-dihydrate (Merck Cat# 1.06580.5000) solution, and 2 parts of 1.2% sodium hydrogen-phosphate-monohydrate (Merck Cat#. 1.06586.2500) were mixed and 1% TTC (Sigma-Aldrich, St. Louis, MO, USA Cat# BCCC3620) were added. The solution was preheated at 37 °C and protected from light before usage. After 30 min, slices were mounted onto objective slides, which were covered on both sides with CitiFluor ^TM^ (science services).

### 4.11. Fixation

Both neuron-oligodendrocyte co-culture and oligodendroglial cell culture were washed three times with PBS to remove cell debris and afterward fixed with 500 µL of 4% paraformaldehyde (PFA) for 15 min at room temperature (RT). Another three steps of washing with PBS followed in order to remove PFA residues. Cells were kept in PBS at 4 °C until staining.

Slices from NG2-CreERT2 td Tomato mice were fixed for immunohistochemistry. To this end, slices were washed 1× with PBS and thereafter fixed with 4% PFA for 15 min. To cover the whole slice with liquid, 1 mL was added underneath the membrane and 1 mL was added on top of the slice and the membrane. Afterwards, slices were washed once with PBS and transferred from the membrane into a new six-well plate filled with 3–4 mL of 30% sucrose (Roth, Karlsruhe, Germany Cat# 4661.2), diluted in PBS. The slices were kept in sucrose for 24–48 h to dehydrate slices for cryoprotection before being frozen in liquid nitrogen.

### 4.12. Embedding of Slices for Frozen Sections

For quantification analysis, 10× serial 10 µm thick coronal sections were obtained from a single coronal slice. To this end, slices were incubated overnight in 30% sucrose for cryoprotection before embedding in Tissue Tek and blocked at −20 °C. Next, a drop of Tissue Tek (Sakura, Finetek, Umkirch, Germany Cat# 4583) was added into a plastic embedding mold (Sakura, Finetek, Umkirch, Germany Tissue-Tek Cryomold Intermediate 15 mm × 15 mm × 15 mm Cat. No. 4566). The slice was transferred with a spatula and was placed on the drop of Tissue Tek. Then, the plastic embedding mold was filled with Tissue Tek, and the position of the slice was adjusted to be planar using a brush. The mold was then shortly transferred into cooled 2-Methylbutane, and 10 µm frozen sections were performed with a cryostate (Leica CM30505). Three frozen serial sections were collected on microscope slides (Superfrost Plus, Thermo Scientific, Waltham, MA, USA Cat# 10149870), and slides were then stored at −20 °C until IHC was performed.

### 4.13. Immunocytochemistry (ICC)

Fixed cells from oligodendrocyte mono cultures were permeabilized with PBS containing 0.01% Triton X-100 (Sigma-Aldrich), and unspecific staining was blocked with 10% normal goat serum or donkey serum (Sigma-Aldrich) for 40 min as established previously [33]. Cells were then incubated with primary antibodies in PBS with 10% normal goat serum or donkey serum overnight at 4 °C using rat anti-myelin basic protein (MBP; 1:250, Bio Rad., Munich, Germany; Cat# MCA409S RRID:AB_325004). Fixed co-cultures were blocked with PBS containing 0.5% Triton X-100 and 2% normal goat serum and then incubated overnight in 0.1% Triton and 2% normal goat serum containing mouse anti-oligodendrocyte transcription factor 2 (Olig2; 1:500, Merck Millipore, Billerica, MA, USA; Cat# MABN50 RRID:AB_10807410) and rabbit anti-Glial Fibrillary Acidic Protein (GFAP; 1:500, DAKO Agilent, Hamburg, Germany; Cat# Z0334 RRID:AB_10013382). For both culture systems, coverslips were washed with PBS 24 h later and then incubated with secondary antibodies in PBS (diluted 1:500) for 2 h conjugated to goat anti-rat Alexa Fluor 488 (ThermoFisher Scientific; Cat# A-11006 RRID:AB_2534074), goat anti-mouse Alexa Fluor 594 (ThermoFisher Scientific; Cat# A11005 RRID:AB_10561507), goat anti-rabbit (ThermoFisher Scientific; Cat# A11037 RRID:AB_10561549), or goat anti-rat (ThermoFisher Scientific Cat# A-11007, RRID:AB_10561522). Nuclei were stained with 4′,6-diamidin-2-phenylindol (DAPI, Roche, Basel, Switzerland). Images (20×; Zeiss Axionplan2 microscope) were captured using the same light intensity and filters for all images to be compared and processed with Axiovision 4.2 software (Zeiss, Jena, Germany; RRID:SciRes_000111). The analysis was performed using Java software Version 1.54e (ImageJ, RRID:nif-0000-30,467/Wright Cell Imaging Facility, RRID:nif-0000-30,471). When ICC was performed on mono-culture, immunopositive cells were counted in nine randomly chosen fields per coverslip. For co-culture experiments, two coverslips were used per condition. The total number of cells per field was determined via DAPI staining. For quantification, the number of immune-positive cells was compared to the total cell number and expressed as percentage [mean ± standard error of the mean (SEM)].

### 4.14. Immunohistochemistry

For immunohistochemistry, the defrosted brain sections were air-dried for 10 min at R.T. and then rehydrated in dH_2_O for 5 min. The microscopy slides with the brain sections were post-fixated in 4% PFA for 5 min and transferred to −20 °C cold acetone for another 5 min, before being washed with first TBS and then TBS-T (TBS containing 0.02% Triton) again for 5 min each. In order to block unspecific staining, slides were blocked with 100 µL 4% BSA, 5% NGS, 0.2% Triton X-100 in TBS for 30 min at R.T. Primary antibodies were diluted in the same solution used for blocking and were incubated overnight at 4 °C. This blocking and antibody solution composition was used for all antibodies except for the goat anti PDGFR-α antibody, where a concentration of 3% BSA and 5% normal donkey serum (NDS) was used, as the secondary antibodies’ host was donkey. Slices were incubated with primary antibodies using the following dilutions: mouse anti-adenomatosis polyposis coli (CC1; 1:250, GeneTex Cat# GTX16794 RRID:AB_422404), rabbit anti-glial fibrillary acidic protein (GFAP; 1:10.000, DAKO Agilent, Hamburg, Germany; Cat# Z0334 RRID:AB_10013382), mouse anti-myelin basic protein (MBP; 1:250, Biolegend, San Diego, CA, USA, Cat# 836504 RRID:AB_2616694), goat anti-platelet derived growth factor receptor alpha (PDGFR-α; 1:250, Neuromics, Minneapolis, MN, USA catalog #GT15150-100 RRID:AB_2737233), goat anti-sex determining region Y-box 10 (Sox10; 1:200, R&D System, Minneapolis, MN, USA, Cat#AF2864; RRID: AB_442208), mouse anti-breast carcinoma amplified sequence 1 (BCAS1; 1:250, Santa Cruz Cat# Cat# sc-136342, RRID:AB_10839529), and rabbit anti-ionized calcium binding adaptor molecule 1 (Iba1; 1:500, WAKO Pure Chemical Corporation, Osaka, Japan; RRID: AB_839504). After 24 h, slices were washed twice in 1xTBS for 5 min each, and secondary antibodies were diluted 1:200 and applied for 2 h at R.T. along with 4′,6-diamidino-2-phenylindol (DAPI, 1:50) in PBS. The following secondary antibodies were used: goat anti-rat Alexa Fluor 488 (1:200, Thermo Fisher Scientific Cat# A-11006, RRID:AB_2534074); goat anti-rabbit Alexa Fluor 488 (1:200, Thermo Fisher Scientific Cat# A-11008, RRID:AB_143165); goat anti-mouse Alexa Fluor 488 (1:200, Thermo Fisher Scientific Cat# A32728, RRID:AB_2633277), and donkey anti-goat Alexa Fluor 488 (1:200, Thermo Fisher Scientific Cat# A11055 RRID:AB_2534102). After this incubation step, slices were washed first in 0.2% TBS-T and subsequently in TBS for 5 min each. Finally, mounting was performed with Fluoromount g (Thermo Fisher Scientific, Waltham, MA, USA). Stained slides were kept at 4 °C until image acquisition.

### 4.15. Data Analysis

The immunostaining was analyzed with a Zeiss Axioplan 2 fluorescence microscope (Zeiss) and the software Axiovision 4.2 (Zeiss). Images of coronal cross-sections within the lesion location Bregma [−0.82]–[−1.22] were taken using 20 x magnification. Regions of interest (ROIs) were defined by an overlay micrograph comprising 4 images (1 image measures 2195 × 1949 pixel or 482 × 428 µm, with an overlay of boxes from 5%) within the penumbra at the border zone between intact and lesioned tissue at the right hemisphere (see Figure 4). All data were verified in this area. The number of cells that were positive for each marker was counted by using the Java software ImageJ Version 1.54e (ImageJ).

### 4.16. Statistical Analysis

Data are shown as mean values ± standard deviation (mean ± SD). GraphPad Prism 7.0.2 (GraphPad Prism, San Diego, CA, USA, RRID: rid_000081) was used for statistics and graphics collection. To assess the absence of Gaussian distribution, the Shapiro–Wilk normality test was used for all data sets. Student’s *t*-test was applied for comparing two groups, and two-way analysis of variance (ANOVA) with Tukey post-test for multiple comparisons was applied to compare three or more groups. For data sets not passing the Shapiro–Wilk normality test, the Mann–Whitney U comparing two groups and the Kruskal–Wallis test with Dunn’s post-test for multiple comparisons of three or more groups were applied. *p* values are defined as follows: * represents *p* ≤ 0.05; ** represents *p* ≤ 0.01; *** represents *p* ≤ 0.001. The absence of asterisks means no statistically significant difference was observed. *n* represents the number of independent experiments.

## 5. Conclusions

Taken together, the study here presented introduces an ex vivo model that has the potential to improve and facilitate stroke research. Furthermore, we could identify parbendazole as a promising treatment to enhance post-stroke recovery.

## Figures and Tables

**Table 1:** (Media used for slice culture).

Medium	Composition	Supplier
Serum-free medium (SFM)	Neurobasal medium 2% B27 supplement 1% N2 supplement 1% L-Glutamine (×100) 0.5% Glucose 1% Antibiotic/antimycotic (×100)	Gibco Cat# 21103-049 Gibco Cat# 17504-044 Gibco Cat#17502-048 Gibco Sigma-Aldrich, Cat# SLBF1738V Sigma-Aldrich, Cat# A5955
Serum-supplemented medium (SSM)	Neurobasal medium 10% Fetal bovine serum (FBS) 0.5% Glucose 1% Antibiotic/antimycotic (×100)	Gibco Cat# 21103-049 Roth, Cat# 8076.2 Sigma-Aldrich, Cat# SLBF1738V Sigma-Aldrich, Cat# A5955

## Data Availability

The data sets used and/or analyzed during the current study are available from the corresponding author on reasonable request.

## Abbreviations

2-DG	2-deoxy-D-glucose
BCAS1	Breast carcinoma amplified sequence 1
BDNF	Brain derived growth factor
BSA	Bovine serum albumin
CC1	Monoclonal antibody anti-adenomatous polyposis coli (APC) clone
CNS	Central nervous system
cPT	Cerebral photothrombosis
DAPI	4′,6-diamidin-2-phenylindol
DMSO	Dimethylsulfoxid
DOAJ	Directory of open access journals
DIV	Days in vitro
FBS	Fetal bovine serum
GFAP	Glial fibrillary acidic protein
HBSS	Hank’s balanced salt solution
ICC	Immunocytochemistry
IHC	Immunohistochemistry
IRB	Institutional Review Board
LD	Linear dichroism
MBP	Myelin basic protein
MDPI	Multidisciplinary Digital Publishing Institute
NaN3	sodium azide
NDS	Normal donkey serum
NG2	Neural/glial antigen 2
NGF	Nerf growth factor
NGS	Normal goat serum
Olig2	Oligodendrocyte transcription factor 2
OL	Oligodendrocyte
OPC	Oligodendroglial precursor cell
OCSC	Organotypic coronal slice culture
PDGFR-α	Platelet-derived growth factor receptor alpha
PDL	Poly-D-lysine
PFA	Paraformaldehyde
PBS	Phosphate buffered saline
ROI	Region of interest
RT	Room temperature
SD	Standard deviation
SEM	Standard error of the mean
SFM	Serum-free medium
SSM	Serum-supplemented medium
SOX10	SRY (sex determining region Y) -Box Transcription Factor 10
T3	Tri-iodo-thyronine
tdT^+^	td-Tomato positive cells
TLA	Three letter acronym
TTC	2,3,5-Triphenyltetrazolium chloride
wt	Wild type
